# Supplementary material for: Fast-Evolving Alignment Sites Are Highly Informative for Reconstructions of Deep Tree of Life Phylogenies
Source: Microorganisms. 2023 Oct 5;11(10):2499. doi: 10.3390/microorganisms11102499 (PMC10609509; doi:10.3390/microorganisms11102499)
Supplement: Supplementary file 1 [file microorganisms-11-02499-s001.zip › microorganisms-2562894-supplementary.pdf]

Supporting Information

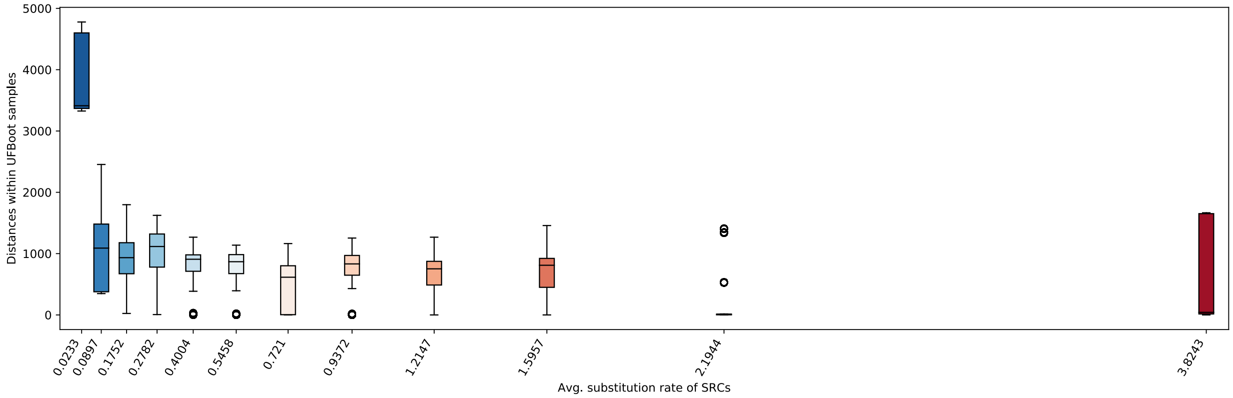

Figure S1. Boxplots representing pairwise RF distances among UFBoot samples within each RSAP from the Hug et al. alignment. Boxplot positions along the X axis represent the average site specific substitution rate of its sites.

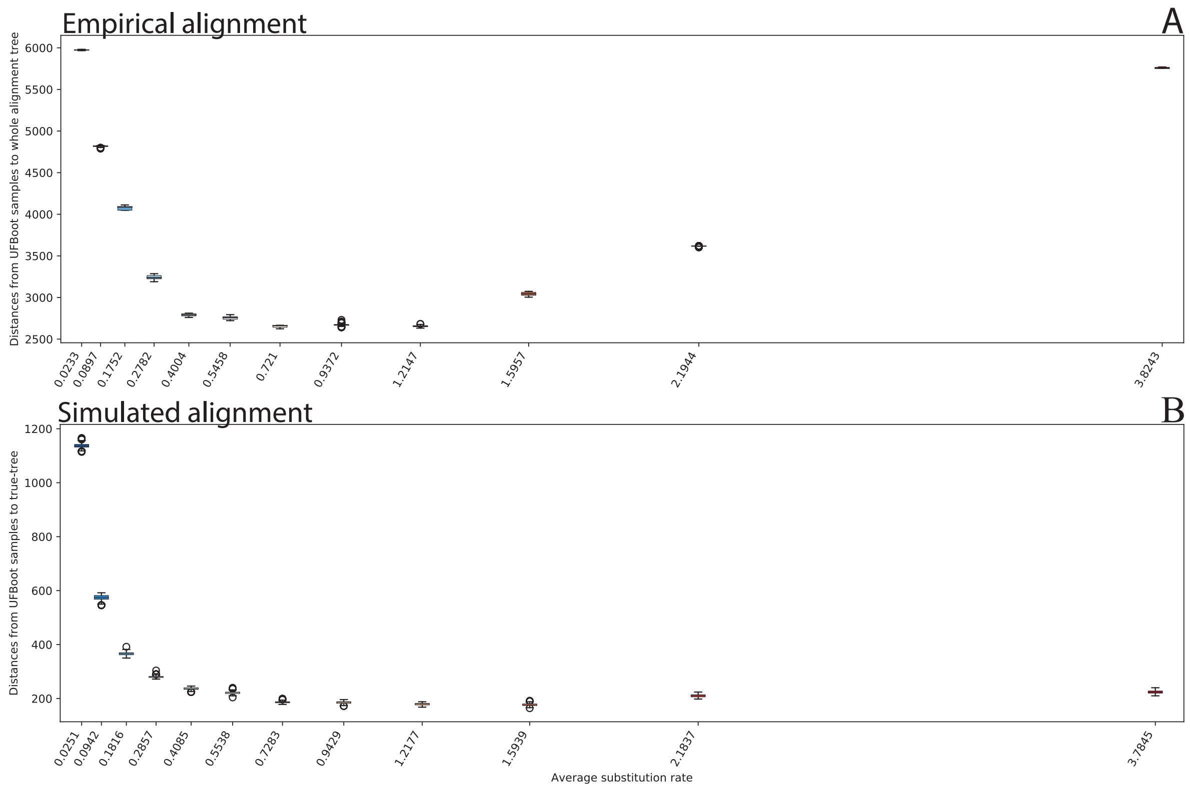

Figure S2. Boxplots representing pairwise RF distances between UFBoot samples from each RSAP versus its reference overall phylogeny. (A) RF distances between UFBoot samples and the Tree of Life topology reconstructed from the whole alignment published by Hug et al.. (B) RF distances between UFBoot samples against the true topology used to generate sequence simulations. Phylogenetic reconstructions of simulated dataset were performed using the same evolution model as its generation, LG+G. Boxplot positions along the X axis represent the average site specific substitution rate of its sites.

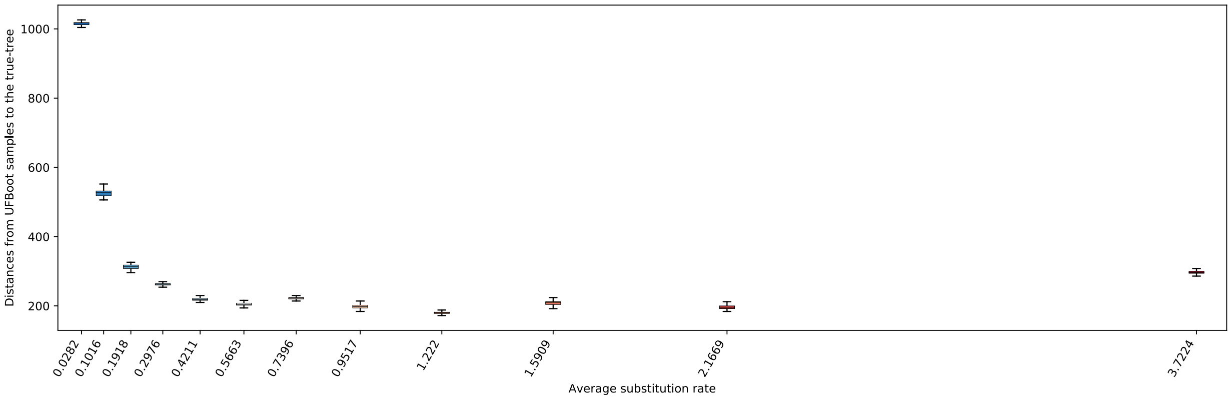

Figure S3. Boxplots representing pairwise RF distances between UFBoot samples of the simulation dataset reconstructed using Dayhoff+G model versus the true topology used to generate sequence simulations. Boxplot positions along the X axis represent the average site specific substitution rate of its sites.

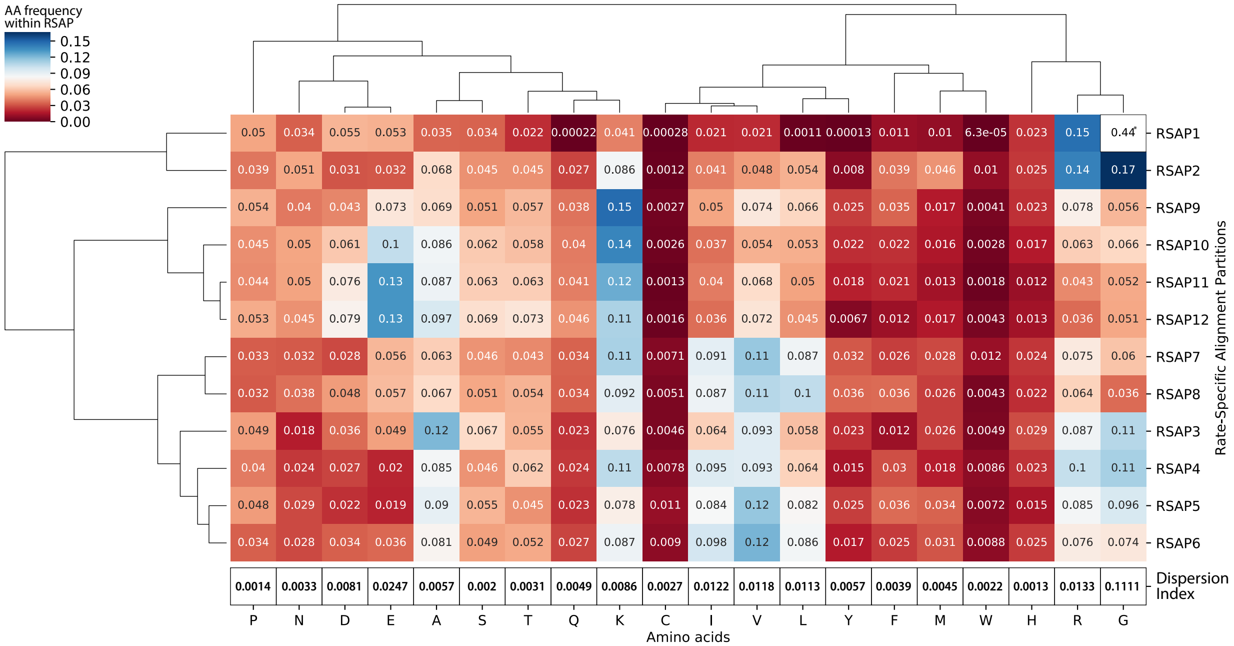

Figure S4. Hierarchical clustering of amino acids (X axis) and RSAPs (Y axis) using amino acid frequency correlations. The Dispersion Index row (defined as $\frac{\sigma^2}{\mu}$) represents how much the frequency of each amino acid varies among RSAPs. Clustering was performed using complete linkage in both axes. *Glycine's ratio within RSAP1 is out of scale to avoid color scale compression of the remaining frequencies.

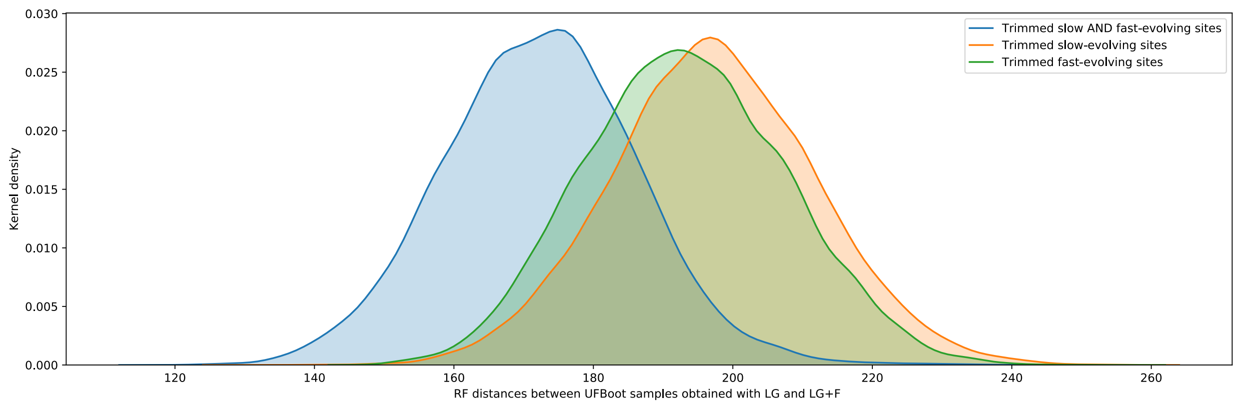

Figure S5. Distribution of RF distances between UFBoot samples obtained using two distinct evolutionary models: LG+G vs LG+F+G. In orange are RF distances between UFBoot samples obtained using the distinct evolutionary models with an alignment partition with trimmed slow-evolving sites (i.e., SRC1 to SRC4). Similarly, in green is the equivalent RF distances obtained with an alignment partition with trimmed fast-evolving sites (i.e., SRC10 to SRC12). Distances obtained using an alignment partition with trimmed both slow and fast-evolving sites are represented in blue.

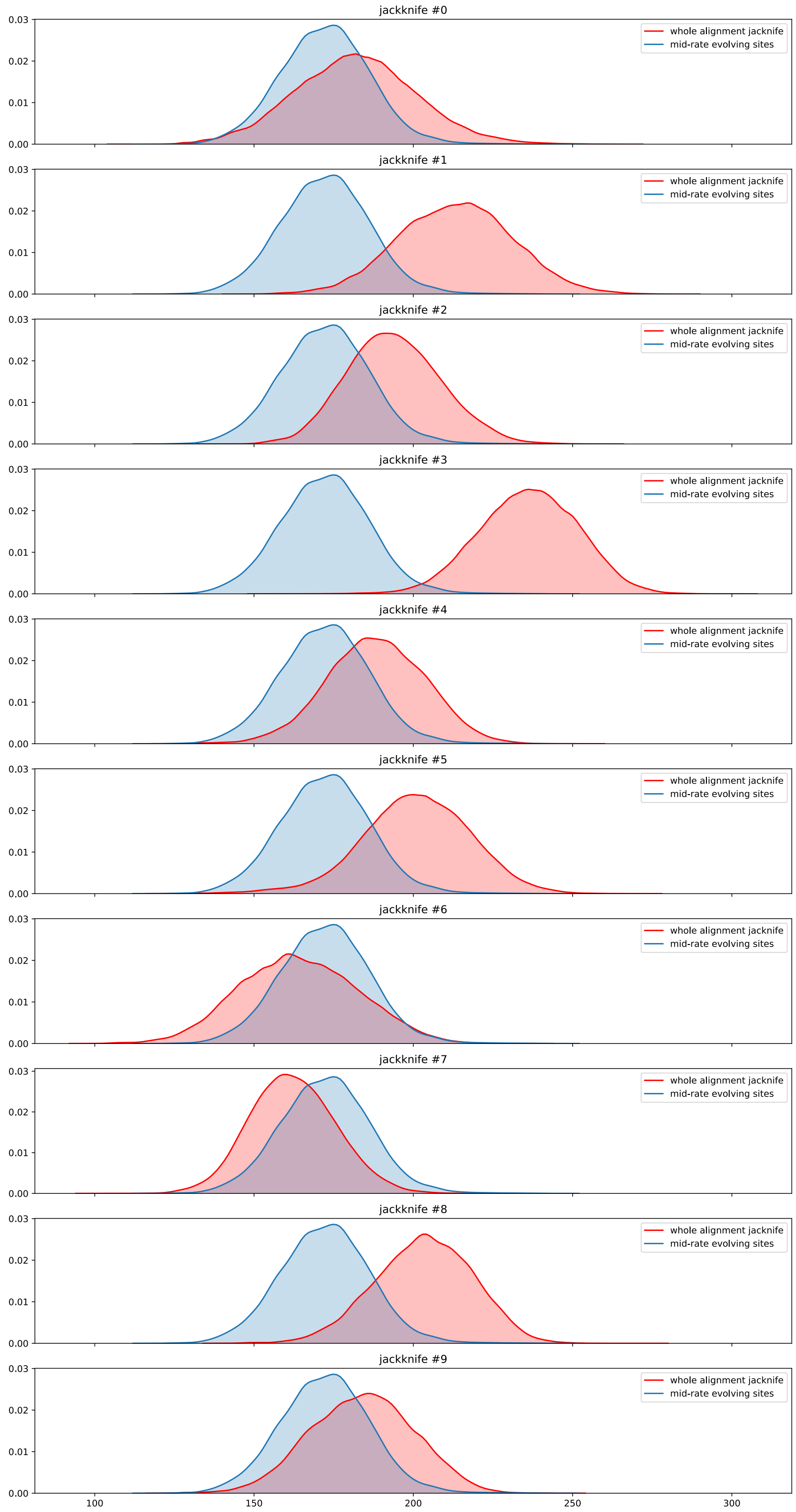

.

Figure S6. Distribution of RF distances between UFBoot samples obtained using two distinct evolutionary models: LG+G vs LG+F+G. Distances obtained using the whole alignment are represented in red, while distances from an alignment partition with trimmed both slow and fast-evolving sites are represented in blue. The whole alignment partition was jackknifed down to 1,420 sites, same length as the trimmed partition. Each subfigure represents a distinct random jackknife of the whole alignment.
